# Bis(benzohydrazide-κ^2^ *O*,*N*′)bis­(nitrato-κ*O*)copper(II)

**DOI:** 10.1107/S1600536809029936

**Published:** 2009-07-31

**Authors:** Elhadj Ibrahima Thiam, Aliou Hamady Barry, Alda Navaza, Pascal Retailleau, Mohamed Gaye, Abdou Salam Sall

**Affiliations:** a Département de Chimie, Faculté des Sciences et Techniques, Université Cheikh Anta Diop, Dakar, Sénégal; b ANBioPhi FRE 3207 CNRS, Université de Paris 13, 74 Rue Marcel Cachin, 93017, Bobigny, France; c ICSN-CNRS, Laboratoire de Cristallochimie, 1 Avenue la Terasse, 91198 Gift sur Yvette, France

## Abstract

In the title compound, [Cu(NO_3_)_2_(C_7_H_8_N_2_O)_2_], the Cu^II^ atom is located on a centre of inversion, and is coordinated by two bidentate benzohydrazide ligands and two monodentate nitrate anions in an axially distorted octa­hedral geometry within an N_2_O_4_ donor set. The crystal structure is stabilized by N—H⋯O and weak N—H⋯N hydrogen bonds.

## Related literature

For related structures, see: Sousa-Pedrares *et al.* (2008 ▶); Despaigne *et al.* (2009 ▶); Hernández-Gil *et al.* (2009 ▶).
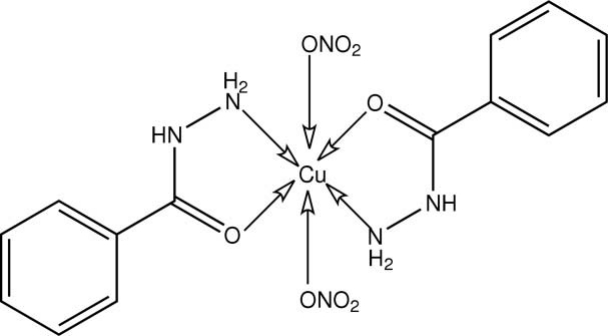


## Experimental

### 

#### Crystal data

[Cu(NO_3_)_2_(C_7_H_8_N_2_O)_2_]
*M* *_r_* = 459.86
Monoclinic, 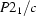

*a* = 10.259 (5) Å
*b* = 10.078 (5) Å
*c* = 9.762 (4) Å
β = 106.85 (1)°
*V* = 966.0 (8) Å^3^
*Z* = 2
Mo *K*α radiation
μ = 1.19 mm^−1^
*T* = 293 K
0.10 × 0.10 × 0.10 mm

#### Data collection

Nonius KappaCCD diffractometer
Absorption correction: none
3237 measured reflections
1768 independent reflections
1278 reflections with *I* > 2σ(*I*)
*R*_int_ = 0.029

#### Refinement

*R*[*F* ^2^ > 2σ(*F* ^2^)] = 0.036
*wR*(*F* ^2^) = 0.094
*S* = 1.05
1768 reflections
145 parameters
3 restraints
H atoms treated by a mixture of independent and constrained refinement
Δρ_max_ = 0.24 e Å^−3^
Δρ_min_ = −0.26 e Å^−3^

### 

Data collection: *COLLECT* (Nonius, 1998 ▶); cell refinement: *DENZO*/*SCALEPACK* (Otwinowski & Minor, 1997 ▶); data reduction: *DENZO*/*SCALEPACK*; program(s) used to solve structure: *SHELXS97* (Sheldrick, 2008 ▶); program(s) used to refine structure: *SHELXL97* (Sheldrick, 2008 ▶); molecular graphics: *PLATON* (Spek, 2009 ▶); software used to prepare material for publication: *SHELXL97*.

## Supplementary Material

Crystal structure: contains datablocks I, global. DOI: 10.1107/S1600536809029936/tk2516sup1.cif

Structure factors: contains datablocks I. DOI: 10.1107/S1600536809029936/tk2516Isup2.hkl

Additional supplementary materials:  crystallographic information; 3D view; checkCIF report

## Figures and Tables

**Table 1:** Hydrogen-bond geometry (Å, °)

*D*—H⋯*A*	*D*—H	H⋯*A*	*D*⋯*A*	*D*—H⋯*A*
N1—H1⋯O4^i^	0.911 (17)	1.94 (2)	2.794 (4)	156 (3)
N1—H1⋯N3^i^	0.911 (17)	2.64 (2)	3.371 (4)	138 (2)
N2—H2*A*⋯O2^ii^	0.929 (18)	2.03 (2)	2.813 (3)	141 (3)
N2—H2*A*⋯O3^i^	0.929 (18)	2.60 (3)	3.186 (3)	122 (2)
N2—H2*B*⋯O2^iii^	0.912 (18)	1.97 (2)	2.834 (3)	159 (3)

Symmetry codes: (i) 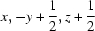; (ii) 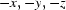; (iii) 
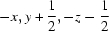
.

**Fig. 1.**
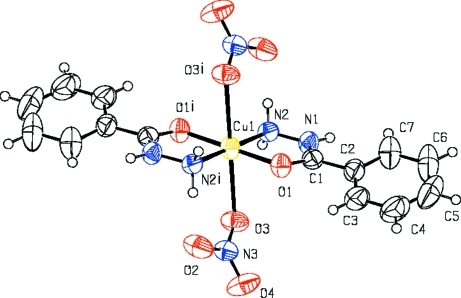
The molecular structure of (I), showing 50% probability displacement ellipsoids and the atom-numbering scheme. Symmetry code: (i) -x, -y, -z

## supplementary crystallographic information

### Comment

The Cu^II^ cation in (I), Fig. 1, is located on a centre of inversion. The Cu^II^ ion is coordinated to two neutral hydrazone molecules functioning as chelating ligands through the amine-N and carbonyl-O atoms. The equatorial bond Cu–O and Cu-N lengths [1.940 (2) and 1.970 (3) Å, respectively] are similar to those observed in related compounds (Sousa-Pedrares et al.<i\>, 2008; Despaigne et al.<i\>, 2009). The remaining coordination positions are occupied by two nitrate-O atoms which are located in apical positions [O1–Cu–O3 = 82.49 (8) °; and Cu–O3 = 2.589 (2) Å]. The axially distorted N_2_O_4_ coordination geometry is consistent with a Jahn–Teller effect (Hernández-Gilet al.<i\>, 2009). In the crystal structure, intermolecular N—H···O and (weak) N—H···N hydrogen bonds interactions link the molecules into a 2-D array (Table 1).

### Experimental

All purchased chemicals and solvents were reagent grade and used without further purification. To a mixture of benzohydrazide (0.2721 g, 2 mmol) and methanol (10 ml) was added dropwise a solution of copper nitrate trihydrate (0.2416 g, 1 mmol) in methanol (10 ml). The resulting green solution was stirred and refluxed for 2 h. The compound was filtered, and slow evaporation of the filtrate gave 0.2930 g (63.7 %) of (I). Analysis: calculated for C_14_H_16_CuN_4_O_8_: C 36.57, H 3.51, N 18.28 %; found: C 36.55, H 3.48, N 18.13. Crystals were obtained from slow evaporation of an ethanol solution of (I).

### Refinement

The H atoms of the NH and NH_2_ groups were located in the Fourier difference maps and refined with N—H = 0.96 (2) Å. The remaining H atoms were placed geometrically and refined in the riding model approximation with C—H = 0.93 Å, and with  *U*_iso_(H) = 1.2*U*_eq_(C)].

### Crystal data

**Table:**

[Cu(NO_3_)_2_(C_7_H_8_N_2_O)_2_]	*F*(000) = 470
*M**_r_* = 459.86	*D*_x_ = 1.581 Mg m^−^^3^
Monoclinic, *P*2_1_/*c*	Mo *K*α radiation, λ = 0.71073 Å
Hall symbol: -P 2ybc	Cell parameters from 1847 reflections
*a* = 10.259 (5) Å	θ = 0.4–25.4°
*b* = 10.078 (5) Å	µ = 1.19 mm^−^^1^
*c* = 9.762 (4) Å	*T* = 293 K
β = 106.85 (1)°	Prism, blue
*V* = 966.0 (8) Å^3^	0.10 × 0.10 × 0.10 mm
*Z* = 2	

### Data collection

**Table:**

Nonius KappaCCD diffractometer	1278 reflections with *I* > 2σ(*I*)
Radiation source: fine-focus sealed tube	*R*_int_ = 0.029
graphite	θ_max_ = 25.4°, θ_min_ = 2.9°
π scans	*h* = −12→12
3237 measured reflections	*k* = −12→11
1768 independent reflections	*l* = −11→11

### Refinement

**Table:**

Refinement on *F*^2^	Primary atom site location: structure-invariant direct methods
Least-squares matrix: full	Secondary atom site location: difference Fourier map
*R*[*F*^2^ > 2σ(*F*^2^)] = 0.036	Hydrogen site location: inferred from neighbouring sites
*wR*(*F*^2^) = 0.094	H atoms treated by a mixture of independent and constrained refinement
*S* = 1.05	*w* = 1/[σ^2^(*F*_o_^2^) + (0.0419*P*)^2^ + 0.3254*P*] where *P* = (*F*_o_^2^ + 2*F*_c_^2^)/3
1768 reflections	(Δ/σ)_max_ = 0.003
145 parameters	Δρ_max_ = 0.24 e Å^−^^3^
3 restraints	Δρ_min_ = −0.26 e Å^−^^3^

### Special details

**Table:**

Geometry. All e.s.d.'s (except the e.s.d. in the dihedral angle between two l.s. planes) are estimated using the full covariance matrix. The cell e.s.d.'s are taken into account individually in the estimation of e.s.d.'s in distances, angles and torsion angles; correlations between e.s.d.'s in cell parameters are only used when they are defined by crystal symmetry. An approximate (isotropic) treatment of cell e.s.d.'s is used for estimating e.s.d.'s involving l.s. planes.
Refinement. Refinement of *F*^2^ against ALL reflections. The weighted *R*-factor *wR* and goodness of fit *S* are based on *F*^2^, conventional *R*-factors *R* are based on *F*, with *F* set to zero for negative *F*^2^. The threshold expression of *F*^2^ > σ(*F*^2^) is used only for calculating *R*-factors(gt) *etc*. and is not relevant to the choice of reflections for refinement. *R*-factors based on *F*^2^ are statistically about twice as large as those based on *F*, and *R*- factors based on ALL data will be even larger.

### Fractional atomic coordinates and isotropic or equivalent isotropic displacement parameters (Å^2^)

**Table:**

	*x*	*y*	*z*	*U*_iso_*/*U*_eq_	
Cu1	0.0000	0.0000	0.0000	0.04486 (19)	
O1	−0.19749 (19)	0.00452 (19)	−0.0567 (2)	0.0488 (5)	
O2	−0.0400 (3)	−0.1377 (2)	−0.3265 (2)	0.0779 (8)	
O3	−0.0372 (2)	0.0668 (2)	−0.2647 (2)	0.0604 (6)	
O4	−0.1549 (3)	0.0048 (2)	−0.4748 (2)	0.0777 (8)	
N1	−0.1582 (2)	0.2195 (3)	−0.0063 (3)	0.0490 (6)	
H1	−0.183 (3)	0.3054 (19)	0.001 (3)	0.058 (9)*	
N2	−0.0173 (2)	0.1913 (2)	0.0322 (2)	0.0431 (6)	
H2A	0.020 (3)	0.212 (3)	0.128 (2)	0.065 (10)*	
H2B	0.023 (3)	0.243 (3)	−0.020 (3)	0.061 (10)*	
N3	−0.0780 (3)	−0.0207 (2)	−0.3550 (3)	0.0492 (6)	
C1	−0.2427 (3)	0.1197 (3)	−0.0488 (3)	0.0466 (7)	
C2	−0.3914 (3)	0.1439 (3)	−0.0875 (3)	0.0544 (8)	
C3	−0.4773 (3)	0.0501 (4)	−0.1710 (4)	0.0732 (10)	
H3	−0.4410	−0.0246	−0.2024	0.088*	
C4	−0.6155 (4)	0.0674 (6)	−0.2074 (4)	0.0973 (14)	
H4	−0.6723	0.0049	−0.2653	0.117*	
C5	−0.6709 (4)	0.1739 (6)	−0.1605 (5)	0.1008 (16)	
H5	−0.7649	0.1841	−0.1857	0.121*	
C6	−0.5871 (5)	0.2671 (5)	−0.0752 (6)	0.1043 (16)	
H6	−0.6248	0.3400	−0.0423	0.125*	
C7	−0.4457 (4)	0.2523 (4)	−0.0379 (5)	0.0804 (11)	
H7	−0.3890	0.3150	0.0198	0.096*	

### Atomic displacement parameters (Å^2^)

**Table:**

	*U*^11^	*U*^22^	*U*^33^	*U*^12^	*U*^13^	*U*^23^
Cu1	0.0462 (3)	0.0307 (3)	0.0588 (3)	−0.0001 (2)	0.0170 (2)	−0.0012 (2)
O1	0.0476 (11)	0.0350 (11)	0.0640 (12)	−0.0019 (10)	0.0166 (10)	−0.0027 (10)
O2	0.146 (2)	0.0381 (13)	0.0555 (13)	0.0204 (14)	0.0385 (14)	0.0072 (10)
O3	0.0891 (17)	0.0414 (12)	0.0477 (12)	−0.0098 (12)	0.0154 (12)	−0.0092 (10)
O4	0.104 (2)	0.0504 (15)	0.0546 (14)	−0.0168 (13)	−0.0155 (14)	0.0068 (11)
N1	0.0501 (15)	0.0358 (14)	0.0573 (15)	0.0064 (12)	0.0097 (12)	−0.0033 (12)
N2	0.0495 (15)	0.0348 (13)	0.0426 (14)	−0.0011 (11)	0.0095 (12)	−0.0008 (11)
N3	0.0673 (16)	0.0380 (16)	0.0437 (14)	−0.0045 (12)	0.0182 (13)	0.0015 (11)
C1	0.0523 (17)	0.0462 (19)	0.0420 (16)	0.0038 (14)	0.0147 (14)	0.0047 (13)
C2	0.0501 (17)	0.060 (2)	0.0540 (18)	0.0083 (16)	0.0171 (15)	0.0102 (16)
C3	0.054 (2)	0.099 (3)	0.062 (2)	0.000 (2)	0.0083 (17)	−0.004 (2)
C4	0.057 (2)	0.150 (5)	0.075 (3)	−0.007 (3)	0.003 (2)	0.005 (3)
C5	0.051 (2)	0.136 (5)	0.111 (4)	0.017 (3)	0.016 (2)	0.051 (3)
C6	0.079 (3)	0.097 (4)	0.153 (4)	0.040 (3)	0.059 (3)	0.039 (3)
C7	0.065 (2)	0.068 (3)	0.114 (3)	0.014 (2)	0.035 (2)	0.011 (2)

### Geometric parameters (Å, °)

**Table:**

Cu1—O1^i^	1.940 (2)	N2—H2B	0.912 (18)
Cu1—O1	1.940 (2)	C1—C2	1.482 (4)
Cu1—N2^i^	1.970 (3)	C2—C7	1.377 (5)
Cu1—N2	1.970 (3)	C2—C3	1.385 (5)
Cu1—O3	2.589 (2)	C3—C4	1.369 (5)
O1—C1	1.261 (3)	C3—H3	0.9300
O2—N3	1.249 (3)	C4—C5	1.355 (7)
O3—N3	1.231 (3)	C4—H4	0.9300
O4—N3	1.233 (3)	C5—C6	1.378 (7)
N1—C1	1.314 (4)	C5—H5	0.9300
N1—N2	1.413 (3)	C6—C7	1.397 (5)
N1—H1	0.911 (17)	C6—H6	0.9300
N2—H2A	0.929 (18)	C7—H7	0.9300
			
O1^i^—Cu1—O1	180.00 (3)	O4—N3—O2	118.7 (3)
O1^i^—Cu1—N2^i^	83.53 (9)	O1—C1—N1	120.2 (3)
O1—Cu1—N2^i^	96.47 (9)	O1—C1—C2	120.4 (3)
O1^i^—Cu1—N2	96.47 (9)	N1—C1—C2	119.4 (3)
O1—Cu1—N2	83.53 (9)	C7—C2—C3	119.7 (3)
N2^i^—Cu1—N2	180.00 (14)	C7—C2—C1	122.2 (3)
O1^i^—Cu1—O3	97.51 (8)	C3—C2—C1	118.0 (3)
O1—Cu1—O3	82.49 (8)	C4—C3—C2	120.0 (4)
N2^i^—Cu1—O3	95.12 (8)	C4—C3—H3	120.0
N2—Cu1—O3	84.88 (8)	C2—C3—H3	120.0
C1—O1—Cu1	112.10 (18)	C5—C4—C3	121.2 (5)
N3—O3—Cu1	116.74 (17)	C5—C4—H4	119.4
C1—N1—N2	117.4 (2)	C3—C4—H4	119.4
C1—N1—H1	125.2 (19)	C4—C5—C6	119.7 (4)
N2—N1—H1	117.4 (19)	C4—C5—H5	120.2
N1—N2—Cu1	106.71 (17)	C6—C5—H5	120.2
N1—N2—H2A	108.4 (19)	C5—C6—C7	120.2 (4)
Cu1—N2—H2A	111 (2)	C5—C6—H6	119.9
N1—N2—H2B	109.6 (19)	C7—C6—H6	119.9
Cu1—N2—H2B	113 (2)	C2—C7—C6	119.2 (4)
H2A—N2—H2B	108 (3)	C2—C7—H7	120.4
O3—N3—O4	121.4 (3)	C6—C7—H7	120.4
O3—N3—O2	119.8 (3)		
			
N2^i^—Cu1—O1—C1	−179.41 (19)	N2—N1—C1—O1	−1.4 (4)
N2—Cu1—O1—C1	0.59 (19)	N2—N1—C1—C2	178.8 (2)
O3—Cu1—O1—C1	−85.07 (19)	O1—C1—C2—C7	157.9 (3)
O1^i^—Cu1—O3—N3	105.7 (2)	N1—C1—C2—C7	−22.2 (4)
O1—Cu1—O3—N3	−74.3 (2)	O1—C1—C2—C3	−19.0 (4)
N2^i^—Cu1—O3—N3	21.6 (2)	N1—C1—C2—C3	160.8 (3)
N2—Cu1—O3—N3	−158.4 (2)	C7—C2—C3—C4	1.9 (5)
C1—N1—N2—Cu1	1.7 (3)	C1—C2—C3—C4	179.0 (3)
O1^i^—Cu1—N2—N1	178.82 (16)	C2—C3—C4—C5	−1.5 (6)
O1—Cu1—N2—N1	−1.18 (16)	C3—C4—C5—C6	0.3 (7)
O3—Cu1—N2—N1	81.82 (16)	C4—C5—C6—C7	0.4 (7)
Cu1—O3—N3—O4	146.4 (2)	C3—C2—C7—C6	−1.2 (5)
Cu1—O3—N3—O2	−34.6 (3)	C1—C2—C7—C6	−178.2 (3)
Cu1—O1—C1—N1	0.2 (3)	C5—C6—C7—C2	0.1 (6)
Cu1—O1—C1—C2	−179.91 (19)		

Symmetry codes: (i) −*x*, −*y*, −*z*.

### Hydrogen-bond geometry (Å, °)

**Table:**

*D*—H···*A*	*D*—H	H···*A*	*D*···*A*	*D*—H···*A*
N1—H1···O4^ii^	0.91 (2)	1.94 (2)	2.794 (4)	156 (3)
N1—H1···N3^ii^	0.91 (2)	2.64 (2)	3.371 (4)	138 (2)
N2—H2A···O2^i^	0.93 (2)	2.03 (2)	2.813 (3)	141 (3)
N2—H2A···O3^ii^	0.93 (2)	2.60 (3)	3.186 (3)	122 (2)
N2—H2B···O2^iii^	0.91 (2)	1.97 (2)	2.834 (3)	159 (3)

Symmetry codes: (ii) *x*, −*y*+1/2, *z*+1/2; (i) −*x*, −*y*, −*z*; (iii) −*x*, *y*+1/2, −*z*−1/2.
